# Supplementary material for: Sociodemographic characteristics as predictors of knowledge regarding mode of transmission of Lymphatic Filariasis among population of Nepal
Source: PLOS Glob Public Health. 2022 Oct 11;2(10):e0000082. doi: 10.1371/journal.pgph.0000082 (PMC10022298; doi:10.1371/journal.pgph.0000082)
Supplement: Attachment — Submitted filename: Response to Reviewers.docx [file pgph.0000082.s001.docx]

**Response to Editor and reviewers**

Dear editor and reviewers,

Thank you very much for your worthy comments we have now revised the manuscript as per your comments and suggestion. We hope the manuscript is much improved than the previous one. Your suggestions and comments will be highly appreciated, please let us know if further revision is required. Once again thank you very much for your support. Please find the comments and response matrix below.

Thank you!

| Comments | Response | Remarks |
| --- | --- | --- |
| 1. Does this manuscript meet PLOS Global Public Health’s [publication criteria](https://journals.plos.org/globalpublichealth/s/criteria-for-publication)? Is the manuscript technically sound, and do the data support the conclusions? The manuscript must describe methodologically and ethically rigorous research with conclusions that are appropriately drawn based on the data presented.  Reviewer #1: Yes  Reviewer #2: Yes | Thank you! |  |
| 2. Has the statistical analysis been performed appropriately and rigorously?    Reviewer #1: Yes  Reviewer #2: Yes | Thank you very much! |  |
| 3. Have the authors made all data underlying the findings in their manuscript fully available (please refer to the Data Availability Statement at the start of the manuscript PDF file)?  The [PLOS Data policy](https://journals.plos.org/globalpublichealth/s/data-availability) requires authors to make all data underlying the findings described in their manuscript fully available without restriction, with rare exception. The data should be provided as part of the manuscript or its supporting information, or deposited to a public repository. For example, in addition to summary statistics, the data points behind means, medians and variance measures should be available. If there are restrictions on publicly sharing data—e.g. participant privacy or use of data from a third party—those must be specified.    Reviewer #1: Yes  Reviewer #2: Yes | Thank you! |  |
| 4. Is the manuscript presented in an intelligible fashion and written in standard English?  PLOS Global Public Health does not copyedit accepted manuscripts, so the language in submitted articles must be clear, correct, and unambiguous. Any typographical or grammatical errors should be corrected at revision, so please note any specific errors here.    Reviewer #1: Yes  Reviewer #2: No | Thank you very much, we have revised the manuscript to reduce the typological and grammatical errors. |  |
| 5. Review Comments to the Author  Please use the space provided to explain your answers to the questions above. You may also include additional comments for the author, including concerns about dual publication, research ethics, or publication ethics. (Please upload your review as an attachment if it exceeds 20,000 characters) | Thanks. We have addressed the comments provided. |  |
| Reviewer #1: Abstract 1: please remove abbreviations from abstract section. 2: methods and materials section please replace ‘’…..was the independent variable’’ to ‘’….were the independent variable’’. 3: result section. ‘’…Radio were significant predictors for knowing LF’’. Is it about knowing LF or knowledge on mode of transmission? Please clarify this sentence. Here also radio is one of the predictors. How? Do you mean availability of radio or radio itself? I think it needs amendment. 4: the authors should clarify what type of data they used. In line number 19, ‘’ Secondary data from Nepal Demographic and 20 Health Survey [NDHS] 201 6 were analyzed.’’. In another section I saw, there is interview process. The authors need to clarify which type of data they used for their research. Primary or secondary? | 1. Thank you, we removed the abbreviations from the abstract.  2. We revised as SDC were the independent variables.  3. Thanks. we meant the exposure of radio and it is one of the significant predictors for knowledge on the mode of transmission of LF after controlling other variables.  4. We used secondary data from NDHS. Actually, NDHS used survey questionnaires for data collection. We mentioned it to clarify. We have revised it. |  |
| Methods and materials 1. Some of abbreviations are written first without described first. So, please check abbreviations and acronyms throughout the document whether it is written in full word first (eg. Line number 94, IFC??). 2. Line number 95-97. The authors said ‘’ some methods have been presented here.’’ And other methodological part is published elsewhere. I did not agree with this concept. Even if it is published elsewhere, you need to discuss/describe every method you used in this research. 3. Line number 98: population and sample: The section is mostly about sampling procedure and study setting. The authors should clarify specifically who is the source and study population in this study. 4. Were you included LF patients in your study or general population? 5. I did not see operational definition for knowledge. Who is translated the questionnaire? 6. What did you use to categorize wealth index? Clarify it in the method section. | 1. We have now presented the full form of IFC: International Finance Corporation.  2. The NDHS is an extensive survey, vague information has been collected during the survey but we presented some portion of the data that we are interested in. Thank you, we revised it.  3. As we have already mentioned that NDHS is an extensive survey so multiple survey tools were used to collect the data. All households were the population. For the knowledge of transmission on LF, the household head was the informants/respondents.  4. No, the NDHS did not focus/select LF patients as a study sample but by default, some respondents might have LF.  5. We added the operational definition of knowledge on the mode of transmission of LF as mosquito bites. In this study, knowledge is measured as awareness of LF and mode of transmission. The questionnaire was translated into the local language by research teams and field enumerators during tools validation and before the data collection.  6. Household's dwellings and assets were assessed to categorize the wealth index. We added it in the method section. |  |
| Result section 1. The result section is clearly written; however, the authors should add numbers before per cent. For example, line number 169 ‘More than two-thirds (69%) of respondents were male.’ Instead you should say ‘‘More than two-thirds, 7581 (69%) of respondents were male’’. 2. Line number 182 and 183 seems conclusion of your finding. So take it to conclusion section. In result section you have to write only the findings only. No need to conclude or discuss about the finding. 3. Table 2: ‘’ How does Lymphatic Filariasis transmit: others’’. Please indicate ‘others’ in the footnote of the table. The content of the table is copy paste of the questionnaire. For example: ‘’How does Lymphatic | 1. Thank you we revised it as per your suggestions.  2. Thank you, we moved it to the conclusion section.  3. There was no specified category in the response of '*Others*' in the report of NDHS. Therefore, it is not possible to mention 'others'. It might be other than the above options. |  |
| Filariasis transmit: through the mosquito bite.’’ . the authors should amend the table to make it more clear. I suggest the following format for table 2. Variables Response N % Heard about LF No Yes Transmission ways of LF through the mosquito bite No Yes from contaminated food No Yes a curse from god No Yes Others* No Yes *indicate others | Thank you we revised it as per your comments. |  |
| 4. Table 3 is not self-explanatory. For example: number in No and Yes column are not clear. I mean is it percent or numbers. If it is percent, show us the % in the yes or no cell. In addition to the percent, the authors should add the number of participants in each yes or no column. Again in the table 3, ‘’Knowledge on Lymphatic Filariasis transmit through a mosquito bite’’ should be replaced to‘’ knowledge on LF transmission’’. I think knowledge on mode of transmission should be operationalized. | 4. Thank you for the critical suggestion! We revised it as No (%), and Yes (%) in table 3 and also change *'knowledge on LF transmission*'. |  |
| 5. Line number 205-216: The narration part should be written before table and show us the source table at the end of each paragraph. Again here while writing the findings the authors should put the number and % together by separating with coma (). Do this for all result section. | 5. Thank you we revised it. |  |
| 6. Line number 221 and 222: change ‘’(aOR = 0.22[0.18 - 0.27], p<0.001)’’ to (AOR=0.22, 95%CI: 0.18-0.27, p=0.001). The authors should present the exact P-value rather than using less than or greater than signs. For some variables the AOR and 95%CI were not narrated. For example, line number 226-227, only p-value was shown. The authors have to revise this section. 7. Table 4: please, can you add p-values for each predictor you have found in Model II? The number in the table looks like copy paste of the SPSS output. Amend the numbers in the table. It is good to say for example ‘0.234’ rather than saying ‘.234’. Model I output is written for only wealth status. For other variables the cells are empty. Can you clarify it? | 6. Thank you we have revised it. We also presented the exact P-value.  7. In the first model we present wealth status only as per our prime interest and in model II we adjusted other variables whether other variables would have influenced or not. We also revised the figure as per your comments. |  |
| Discussion The discussion section is not well written. The authors did not discuss major findings in the manuscript. Even though the result is written in good manner, I am not happy with the discussion section. Please discuss pertinent findings in this section including your assumptions why the difference between your result and other studies happen. Is the difference because of population or sample size or geography of the study setting or other? In your study many predictors were significantly associated with knowledge on mode of LF transmission. But these factors should be discussed separately by comparing with other related researches. In addition, I have seen that two different factors were discussed in one paragraph. This is not correct. Line number 292 and 293 ‘’ Both results showed different findings compared to 293 this study.’’ What is your finding?  Reviewer #2: The paper by Adhikari et al. reports the results of a large study assessing the transmission knowledge of lymphatic filariasis, an endemic disease in Nepal. | Thank you very much we have added the major findings as per the study objective in the last sentence of first paragraph in the discussion section.  Similarly, we have also discussed each issues separately in the discussion section. |  |
| The study is based on a large cross-sectional study in a large population, using a well-executed sampling design. The number of population included allows for accurate estimates. However, while the sampling was very well detailed, the description of the sample size is missing.  The main result concerns the economic status of the family, represented by the head of the family. The results clearly show that wealthier people are better informed about the mode of transmission, with a dose response effect.  On the form, probably the English could be improved on the whole document. In addition, there are a lot of errors of editing... : type, no space (line 246), double space; references between two points (line 200), aOR is not defined before using the acronym (line 221).... so there is a lot of form work to be done.  While the discussion does present the equivalent results from other countries, it lacks a discussion of the usefulness of this result. How to explain this result in Nepal, what to do with this result in terms of public health policy. A short paragraph on this aspect should be added, which only lines 316-318. | Sample size is presented at the end of the population and sample section as .."Altogether 11, 473 households were selected as sample size, but the information was obtained from 11,040 household heads."  Thank you very much the manuscript is now edited by professional English language editor  We have edited the line space, word space. OR and AOR are defined at the first sentence under multivariate analysis (page 12).  Thank you! we added it as 'Based on the results and discussion, it can be suggested for policy makers and planners that awareness related programme on LF should be focused on the people having low economic condition'. |  |
