# Supplementary material for: Sociodemographic characteristics as predictors of knowledge regarding mode of transmission of Lymphatic Filariasis among population of Nepal
Source: PLOS Glob Public Health. 2022 Oct 11;2(10):e0000082. doi: 10.1371/journal.pgph.0000082 (PMC10022298; doi:10.1371/journal.pgph.0000082)
Supplement: Attachment — Submitted filename: Response to Reviewers.docx [file pgph.0000082.s002.docx]

**Response to Editor and reviewers**

Dear editor and reviewers,

Thank you very much for your worthy comments we have now revised the manuscript as per your comments and suggestion. Please let us know if further revision is required. Once again thank you very much for your support. Please find the comments and response matrix below.

Thank you!

| Comments | Response | Remarks |
| --- | --- | --- |
| 1. Please update your online Competing Interests statement. If you have no competing interests to declare, please state: “The authors have declared that no competing interests exist.” | Thank you very much. We revised it as per the suggestion. We added 'Competing Interest' just above the Reference section. | Page 18 |
| 6. Review Comments to the Author  Please use the space provided to explain your answers to the questions above. You may also include additional comments for the author, including concerns about dual publication, research ethics, or publication ethics. (Please upload your review as an attachment if it exceeds 20,000 characters)  Reviewer #1: My concerns on the manuscript are adequately addressed.  Reviewer #3: The authors appropriately revised the paper based on the initial review. The data analysis method could have elaborated the variable selection criteria used to determine the sociodemographic variables retained in the final multivariable model II. Also, it could be interesting to know how potential collinearity issues were addressed, if any, especially for the variables: having TV vs. Radio, and Province vs. Place of residence. | Thank you very much for valuable comments. We revised it in the **data processing and analysis** section.  We revised as:  We assessed the variables to determine the multi-collinearity among the variables before adjusting the variables in multivariate analysis (in Model II), and found no multi-collinearity issues among the variables. | Page 7 |
|  |  |  |
