# Supplementary material for: Sociodemographic characteristics as predictors of knowledge regarding mode of transmission of Lymphatic Filariasis among population of Nepal
Source: PLOS Glob Public Health. 2022 Oct 11;2(10):e0000082. doi: 10.1371/journal.pgph.0000082 (PMC10022298; doi:10.1371/journal.pgph.0000082)
Supplement: Attachment — Submitted filename: Response to Reviewers.docx [file pgph.0000082.s003.docx]

**Response to Editor and reviewers**

Dear editor and reviewers,

Thank you very much for your worthy comments we have now revised the manuscript as per your comments and suggestion. Please let us know if further revision is required. Once again thank you very much for your support. Please find the comments and response matrix below.

Thank you!

| Comments | Response | Remarks |
| --- | --- | --- |
| This manuscript has received many rounds of reviews and recommendations for revision. Despite this, I have observed that the title would benefit from minor modification. Consider the title to read; "Sociodemographic Characteristics as predictors of Knowledge regarding mode of transmission of Lymphatic Filariasis among population of Nepal", since the outcome variable in the study is Knowledge regarding mode of transmission of Lymphatic Filariasis. | Thank you!  We revised it as per suggestion. | Title |
| The abstract in the Result section in lines 26-27 should be: "Findings showed that only 28 percent of household-heads correctly identified the mode of transmission of Lymphatic Filariasis in the study." | Thank you very much!  Revised accordingly. | Lines 28-29 |
| English rendering in Lines 32-34 should be clearly expressed. | Thank you! Revised as:  Variables: wealth status, sex, residence, eco-belts, possessing bed nets, and Radio appeared as significant predictors for knowledge on the mode of transmission of Lymphatic Filariasis. | Lines 32-34 |
| 6. Review Comments to the Author  Reviewer #3: The authors have addressed all the reviewers' concerns from the previous rounds of reviews.  As much as I recommend the publication of this manuscript, I would like to authors to consider two things:  1. The 2016 Nepal Demographic and Health Survey (NDHS) used sampling weights, and like most population surreys, it allows the sample to be statistically representative of the population. So I do not know if the authors had a reason for leaving out sample weighting in their analyses. Are the reported prevalence weighted or unweighted?  2. Publications have reported the unreliability of p-value especially as a limitation when you have a large sample size, where majority of the bivariate analysis are 'forcefully' significant. Hence, authors should consider other statistical indicators used to support the p-value, such as change-in-estimate etc. | 1. We analysed the weighted sample. The reported prevalence are weighted. We added it at **Data processing and analysis** under the **Materials and methods** section. 2. The survey was not so big but tried to make national representatives. Most of the articles presented on the different issues with reference to NDHS are presented in that way. We followed them to present. We performed multivariate analysis for further identification of the variables that can predict. We thought that multivariate analysis might check the bivariate analysis. Some related articles for reference:   <http://doi.org/10.25133/JPSSv302022.023>  [**https://doi.org/10.1002/hpm.3285**](https://doi.org/10.1002/hpm.3285)  https://www.jnhrc.com.np/index.php/jnhrc/article/view/3738 | Page 7 |
